# Prevalence and Treatment Utilization of Patients Diagnosed with Depression and Anxiety Disorders Based on Kentucky Medicaid 2012–2019 Datasets

**Published:** 2022-06-30

**Authors:** Yuchen Han, Haojiang Huang, Riten Mitra, Huirong Hu, Subhadip Pal, Craig J McClain, K.B. Kulasekera, Maiying Kong

**Affiliations:** 1 Department of Bioinformatics and Biostatistics, University of Louisville School of Public Health and Information Science, Louisville, KY, United States; 2 Department of Psychiatry and Behavioral Sciences, University of Louisville School of Medicine, Louisville, KY, United States; 3 Department of Pharmacology and Toxicology, University of Louisville School of Medicine, Louisville, KY, United States; 4 Department of Medicine, Division of Gastroenterology, Hepatology and Nutrition, University of Louisville School of Medicine, Louisville, KY, United States; 5 Robley Rex Louisville VAMC, Louisville, KY, United States

**Keywords:** Depressive disorders, Anxiety disorders, Treatment, utilization

## Abstract

**Objectives:**

To examine the prevalence and treatment utilization of patients diagnosed with Depression and Anxiety Disorders (DAD) based on Kentucky Medicaid 2012–2019 datasets.

**Methods:**

The study was based on Kentucky Medicaid claims data from 2012 through 2019 for patients 14 years and older. We constructed yearly patient-level databases using ICD_9 CM and ICD_10 CM codes to identify the patients with DAD, using the Current Procedure Terminology (CPT) codes to identify individual psychotherapy and group psychotherapy and using the National drug codes to categorize pharmacotherapy. Based on these data, we constructed summary tables that reflected the trends in prevalence of DAD across eight Kentucky Medicaid regions and for different demographic subgroups. Next, we implemented logistic regression on the constructed yearly patient-level data to formally assess the impact of risk factors and treatments on the prevalence of DAD. The potential risk factors included age, gender, race/ethnicity, geographic characteristics, comorbidities such as alcohol use disorder and tobacco use.

**Results:**

The prevalence of DAD increased from 30.84% in 2012 to 36.04% in 2019. The prevalence of DAD was significantly higher in patients with the following characteristics: non-Hispanic white, females, aged between 45 and 54 years old, living in rural areas, having alcohol use disorder, and using tobaccos. Other than 2013, the utilization of pharmacotherapy maintained at about 62%. The utilization of psychotherapy increased over years from 24.4% in 2012 to 36.5% in 2019. Overall, the utilization of any treatment slightly increased from 70.9% in 2012 to 73.3% in 2019 except a drastic decline in 2013 due to the reduction of benzodiazepine prescription. Patients being whites, females, and living in rural areas were more likely to use pharmacotherapy, and patients living in rural areas were less likely to use psychotherapy than those residing in urban areas.

**Conclusion:**

The prevalence of DAD has increased over time from 2012 to 2019. The utilization of pharmacotherapy maintained at 62% over eight years except 2013, and the utilization of psychotherapy has steadily increased over time.

## INTRODUCTION

Depression and Anxiety Disorders (DAD) are the two most common mental illnesses in the United States (US). Major depressive disorder, a sub-category of depressive disorders, has been ranked as the third leading disease burden worldwide in 2008 by the World Health Organization (WHO) [1]. Furthermore, WHO projected that this disease will rank first by 2030 [1]. The diagnosis of a major depressive order entails an individual having the following symptoms: persistently low or depressed mood, anhedonia or decreased interest in pleasurable activities, feelings of guilt or worthlessness, lack of energy, poor concentration, appetite changes, psychomotor retardation or agitation, sleep disturbances, or suicidal thoughts [2].

Based on the statistics published by the National Alliance on Mental Illness (NAMI) in 2019 [3], the annual prevalence of major depressive episode and anxiety disorders were, respectively, 7.8% and 19.1% among US adults. During 2017–2019, the annual average prevalence of past-year serious mental illness in Kentucky was 6.2% which was higher than the national average (4.8%). Statistics show that 46.0% of adults with mental illness in Kentucky received treatment during 2017–2019 compared to a national rate of 43.6% [4]. People with mental illness do not only have a higher risk of developing cardiovascular and metabolic diseases than the general population, but also experience higher risk of unemployment, leading to more severe financial struggles and poorer health out comes[5].

From SAMHSA [6], Kentucky ranked among one of the top states in the past year of major depressive episodes among people aged 18–25, in the past year serious mental illness among people aged 26 and older, and in the past year prescription pain relief disorders. People insured by Medicaid include eligible low-income adults, children, pregnant women and people with disabilities [7], which usually have an economic disadvantage and are more vulnerable to suffer from DAD. Depression and anxiety in turn worsen their socioeconomic conditions [8]. It is important to identify the geographical regions with higher prevalence of DAD as well as to identify the risk factors of DAD so that some targeted actions could be taken. It is also important to examine treatment modality for patients with DAD and factors associated with utilization of different treatments for DAD. In this article, we focused on quantitative analyses of four major aspects of the DAD problem: DAD prevalence, its geographical distribution, its associated risk factors, and treatment utilizations.

## METHODOLOGY

### Data set and study sample

Data was collected from the Kentucky Medicaid database from 1/1/2012 – 12/31/2019 and included patients 14 years and older. The database included medical claims containing beneficiary identification (ID) number, demographics and geographic information, the International Classification of Diseases (ICD) 9th edition and 10th edition Clinical Modification (ICD-9-CM and ICD-10-CM) codes, the ICD-9-CM procedure codes, Healthcare Common Procedure Coding System (HCPCS) procedure codes and 11-digit National Drug Code (NDC). Yearly segmented datasets were created by linking all claim records, diagnosis codes, procedure codes and drug codes for each patient via the patient’s unique beneficiary ID number. The yearly patient-level dataset included patients’ demographic information (e.g., age, gender, race, and ethnicity), geographic information (e.g., medical region and urban/rural area), diagnoses of interest (e.g., DAD), and treatment information (e.g., pharmacologic treatment and psychosocial therapies). The University of Louisville Institutional Review Board and Kentucky Cabinet for Health and Family Services (KCHFS) reviewed and approved the protocol. A data use agreement with KCHFS Authority permitted the access to the Medicaid database.

### Outcome variable

The primary outcome variable was a binary variable on whether a patient was diagnosed with DAD (i.e., at least one diagnosis code of DAD) in a specified year. A patient was claimed to have DAD if the person had at least one of the following ICD-9-CM and ICD-10-CM diagnosis codes for depressive disorders (ICD-9-CM: 296.2x, 296.3x, 300.4, 311 or ICD-10-CM: F32.x, F33.x, F34.1) or anxiety disorders (ICD-9-CM Codes:293.84, 300.x, 309.81 or ICD-10-CM: F06.4, F40.x, F41.x, F42.x, F44.9, F45.5, F45.6, F45.7,F45.8, F48.8, F48.9, F99, R45.2) (Table S1). The secondary outcome variable was to characterize the treatment utilization for patients diagnosed with DAD. To categorize the pharmacotherapies for patients with DAD, we identified whether a patient with DAD received any FDA approved medications for DAD treatment. These medications based on their drug prescription were classified into seven major categories (Table S2) [9]: (1) Selective Serotonin Reuptake Inhibitors (SSRIs), including fluoxetine, citalopram, sertraline, paroxetine and escitalopram; (2) Serotonin-Norepinephrine Reuptake Inhibitors (SNRIs), including venlafaxine, duloxetine and desvenlafaxine; (3) TriCyclic Antidepressants (TCAs), including amitriptyline, amoxapine, clomipramine, desipramine, doxepin, imipramine, nortriptyline, protriptyline and trimipramine; (4) Tetracyclic antidepressants including mirtazapine; (5) Benzodiazepines, including diazepam, clonazepam, lorazepam and alprazolam; and (6) Bupropion and (7) Hydroxyzine. To examine the utilization of psychosocial therapies for patients with DAD, we created a dichotomous indicator for psychotherapy determined by the Current Procedural Terminology (CPT) codes and classified into individual psychotherapy and group psychotherapy (Table S3) [10]. We further identified whether a patient ever received any treatment (i.e., psychotherapy or pharmacotherapy) in that year.

### Covariates

We obtained the patients’ demographic and geographic characteristics, including age (categorized as<24, 25–34, 35–44, 45–54, 55–64, and >65), gender (female versus male), race/ethnicity (categorized as non-Hispanic white, non-Hispanic black, Hispanic, non-Hispanic other and non-Hispanic missing), Rural-Urban Continuum (RUC) codes, Alcohol Use Disorder (AUD) and tobacco use. Based on the RUC code, a binary variable was created to indicate whether a patient resided in a urban area (RUC codes of 1–3) or a rural area (RUC codes of 4–9). These rural-urban continuum codes were developed by the Department of Agriculture [11] for county-level classification that classified counties by the population size and degree of urbanization and adjacency to a metro area. We also included a geographical variable for the medical region where a patient resided. There were 8 medical regions, and these regions covered all 120 counties across the state of Kentucky [12]. These covariates were identified using the diagnosis codes listed in Table S1 in the Supplement.

### Statistical methods

We first calculated the prevalence of DAD and its subcategories (i.e., depressive disorder, and anxiety disorder) among Kentucky Medicaid patients who had claims from 01/01/2012 to 12/31/2019 (Table 1).

We stratified the patients who were diagnosed with DAD in 2019 into different groups based on patients’ characteristics (Table 2). Here χ^2^ test statistics was applied to examine the association between each covariate and DAD status. We then applied logistic regression models to formally identify risk factors which were significantly associated with the prevalence of DAD. Odds Ratio (OR) and its 95% Confidence Interval (CI) were reported for each covariate (Table 2). We carried out the same analysis for patients who had claims in the 2012 Kentucky Medicaid database, and the results were reported in Table S4 in the supplementary materials.

We also examined treatment utilization for patients diagnosed with DAD. The treatment included pharmacotherapy and/or psychotherapy, where pharmacotherapy included seven different types of medications (Table S2), and psychotherapy included individual and group psychotherapy (Table S3). Different types of treatment utilization for patients diagnosed with DAD were reported for each year over the period from year 2012 to year 2019 (Table 3), where the number of patients and the percentage of patients receiving each specific type of medication or psychotherapy among those diagnosed with DAD were reported. We also examined factors which were associated with treatment utilization based on Kentucky Medicaid 2019 dataset (Table 4), where treatment was classified as medication, psychotherapy, or any of those. All data management and analyses were carried out in the statistical analysis software R. A test statistic was claimed to be significant if the p-value was less than 0.05.

## RESULTS

### Prevalence in Depression and Anxiety Disorder (DAD)

From 2012 through 2019, the prevalence of DAD slightly decreased in 2014 but then steadily increased over years from 2015 to 2019. Among patients diagnosed with DAD in 2019, 80.1% had anxiety disorders, 62.7% had depressive disorders, 7.1% had AUD, and 48.1% used tobacco (Table 1). The prevalence of AUD among those with DAD had increased from 4.8% in 2012 to 7.1% in 2019, which was significantly higher than the prevalence of AUD in the Medicaid population ranging from 2.6% in 2012 to 4.2% in 2019. The prevalence of tobacco uses for patients with DAD ranged from 37.1% in 2012 to 48.1% in 2019, which were also significantly higher than the prevalence of tobacco uses in the Medicaid population ranging from 11.4% in 2012 to 17.5% in 2019.

**Figure 1:**
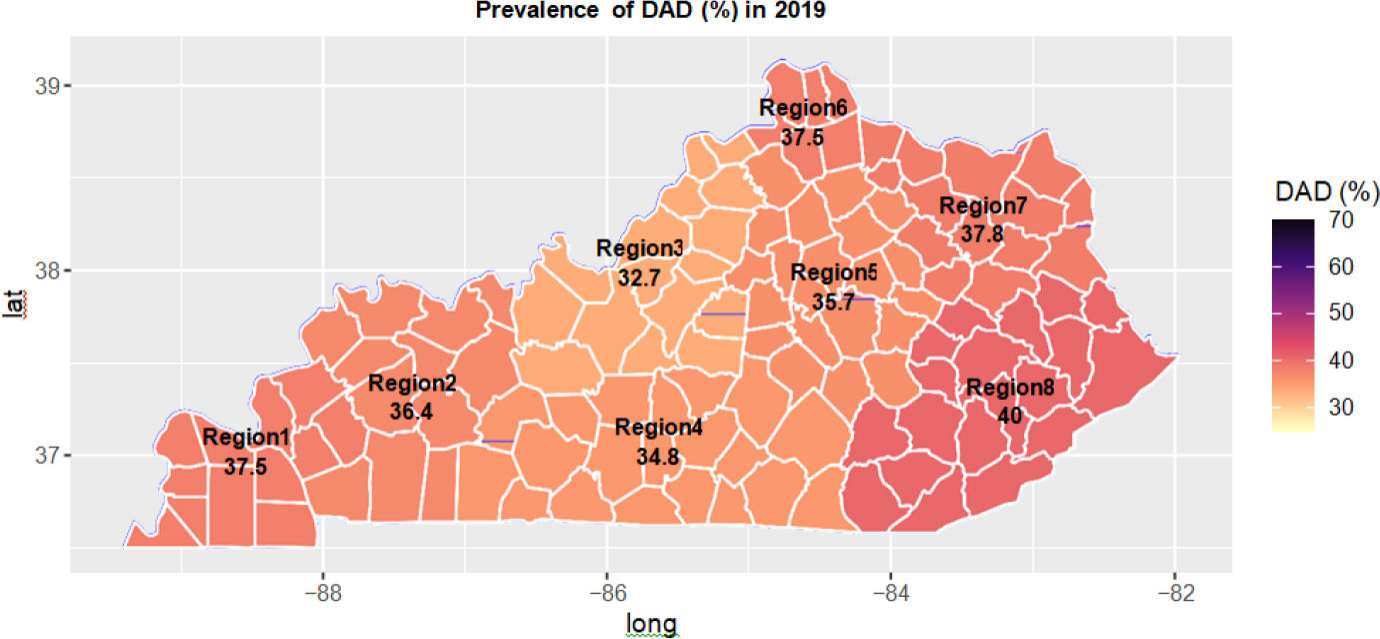
Geographic distribution of prevalence of DAD (Panel A1: prevalence of DAD in 2019).

**Figure 2:**
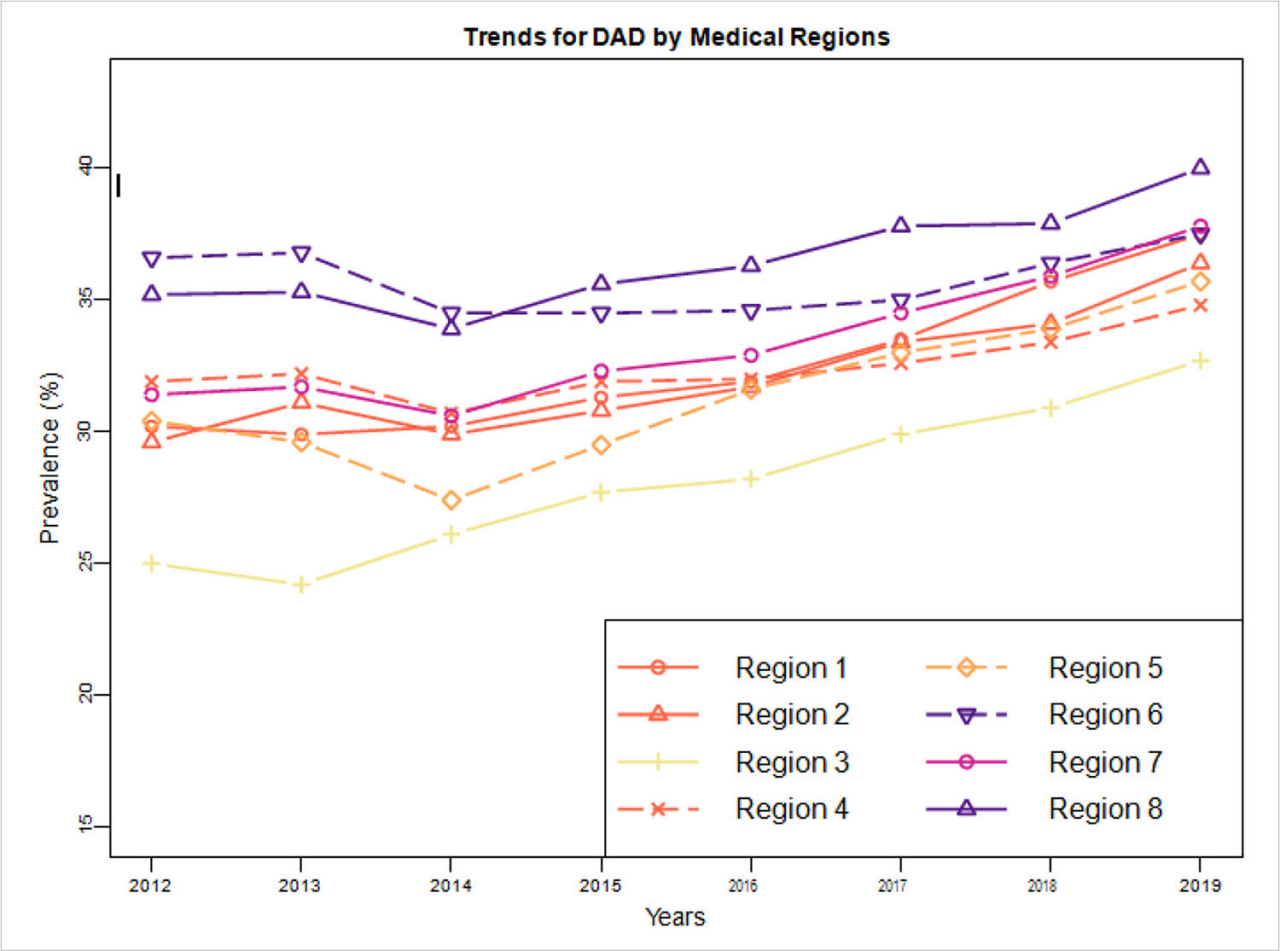
Trends of DAD prevalence over Kentucky eight different medical regions.

Figure 1 showed the geographic distribution of prevalence of DAD over eight Kentucky medical regions in 2019, and Figure 2 showed the trends of DAD prevalence over the years 2012–2019 for each one of eight medical regions [12]. From Figure 1, Region 8 in the eastern Kentucky Appalachian area had the highest DAD prevalence rate (40.0%), followed by Region 6 in the Northern Kentucky (37.8%), and Region 3 in the Louisville area had the lowest DAD prevalence rate (32.7%). The geographic patterns were similar but on a smaller scale based on 2012 data (Figure S1).

From Figure 2, Region 6 had the highest DAD prevalence rate (about 35%) between 2012 to 2014, and Region 8 passing Region 6 had the highest DAD prevalence rate between 2015 to 2019, reaching 40% in 2019. Region 3 had the lowest DAD prevalence rate across all the eight years 2012–2019. The DAD prevalence rates in each one of the eight medical regions increased over time (Figure 2).

### Risk factors for DAD prevalence

**Figure 3:**
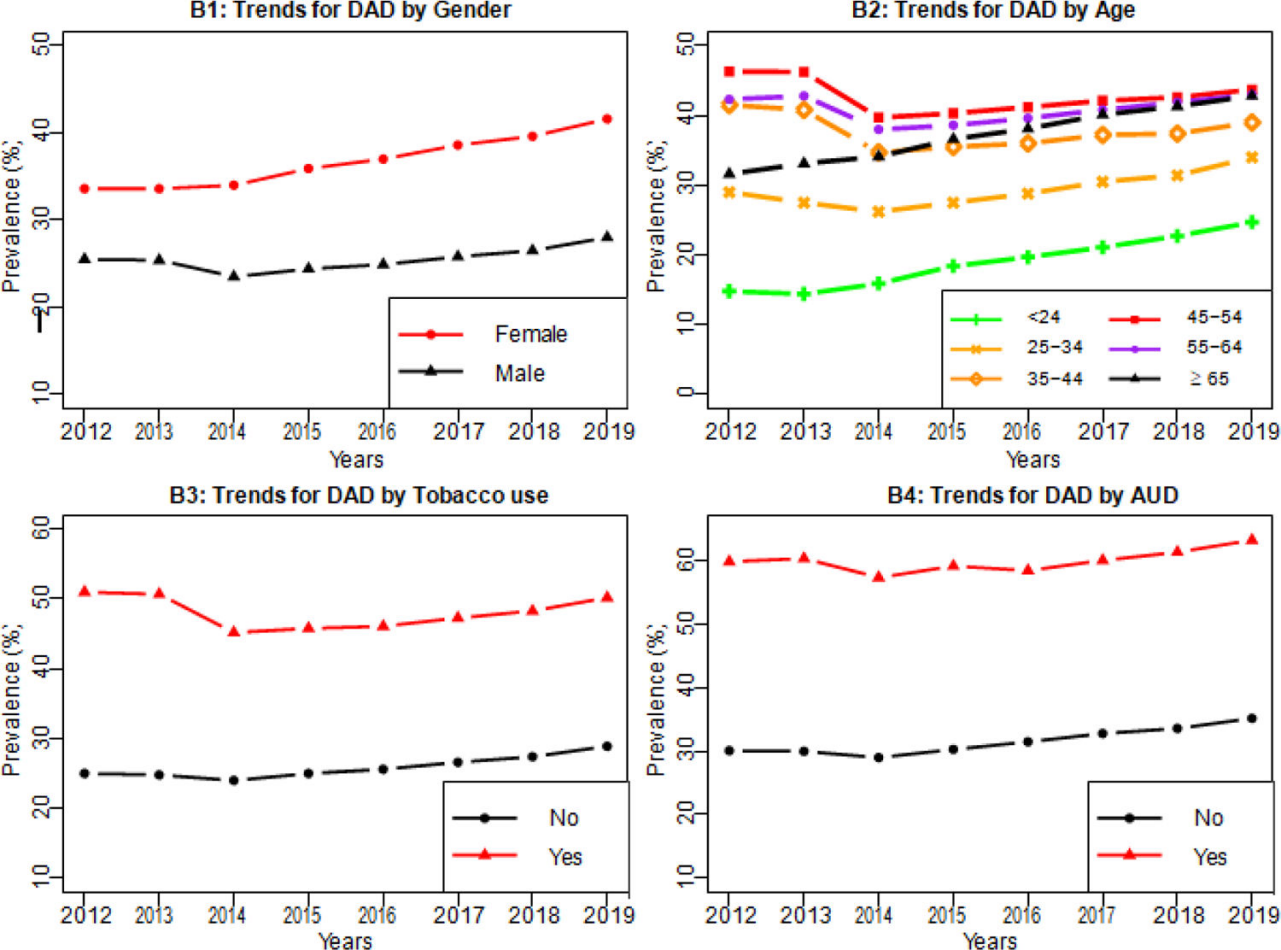
Trends of DAD stratified by risk factors (Panel B1: Gender; Panel B2: Age; Panel B3: Tobacco use; Panel 4: AUD).

We examined the potential risk factors for DAD, which included age, race/ethnicity, gender, geographic area, and the comorbidity conditions such as AUD and tobacco use. From Figure 3, the prevalence of DAD had increased over the years from 2012–2019 for each level of the risk factors. The prevalence of DAD for females were 33.6% in 2012 and increased to 41.9% in 2019, and the prevalence of DAD for males were 25.5% in 2012 and increased to 28.2% in 2019 in (Figure 3B1). The prevalence of DAD for the youngest group (age<24) was the lowest, the prevalence of DAD increased as age increased and reached the highest in the age group of 45–54 years old, and the DAD prevalence slightly dropped for age groups 55–64 and ≥ 65 but was quite close to the highest (Figure 3B2). The prevalence of DAD for tobacco users was 20% higher than non-tobacco users (Figure 3B3). Although the DAD prevalence for patients with tobacco use slightly declined from 51% in 2012 to 45.5% in 2014, it steadily increased to 50.2% in 2019. The prevalence of DAD for non-tobacco users did not change significantly during 2012–2015 (25% in 2012 to 25.1% in 2015) but increased steadily thereafter (26.4% in 2016 to 28.9% in 2019). The prevalence of DAD for patients with AUD was 30% higher than patients without AUD (Figure 3B4).

We applied a logistic regression model to examine whether these risk factors were significantly associated with the prevalence of DAD using 2012 data (Table S4) and 2019 dataset (Table 2) respectively. The results did indicate that those factors were significantly associated with the prevalence of DAD. Non-Hispanic whites had the highest prevalence of DAD (39.4% in 2019), while Hispanic and non-Hispanic blacks had the prevalence of DAD as 19.2% and 23.7% respectively in 2019, with odds ratios of 0.482 (95% CI (0.464, 0.501)) and 0.488 (95% CI (0.480, 0.495)) compared to non-Hispanic whites (Table 2).

The DAD prevalence increased as age increased but plateaued at the aged 45 and above in Table 2, males were 51.6% less likely to have mental disorder than females (OR: 0.484, 95% CI (0.480, 0.489)) in 2019. The DAD prevalence was higher in rural areas than in urban areas (38.3% versus 34.2%) in 2019, with an odds ratio of 1.085 and 95% CI (1.075, 1.096). The DAD prevalence was consistently higher among patients with AUD versus without AUD (63.3% versus 35.2%) in 2019, with an OR of 2.84 and 95%CI (2.78, 2.91) (Table 2). Patients with tobacco use had significantly higher odds of having DAD than those without using tobacco (OR: 2.19, 95%CI: (2.17, 2.21)) in 2019 (Table 2). The analyses based on year 2012 data revealed similar patterns for the risk factors (Table S4) while the prevalence rates in 2012 were lower than those in 2019 (Table S5).

### Trends and patterns of treatment utilization for DAD

We first examined the utilization of seven types of pharmacotherapies, two types of psychotherapies, and the utilization of any one of the treatments for patients diagnosed with DAD.

**Figure 4:**
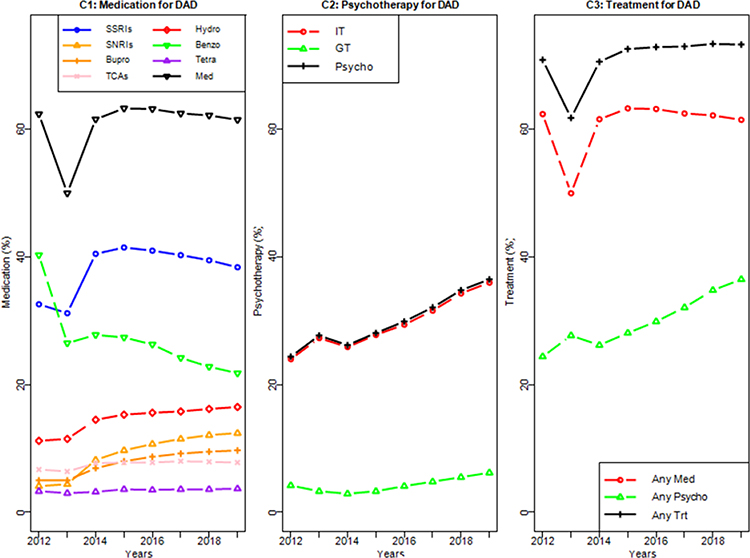
Treatment utilization for patients with depressive and anxiety disorders (DAD) based on 2012–2019 Kentucky Medicaid database (Panel C1: Medication; Panel C2: Psychotherapy; Panel C3: Treatment).

The results from Table 3 and Figure 4C1 clearly showed that (1) Benzodiazepine was the top prescribed medication with 40.3% utilization rate in 2012, which dropped to 26.3%~27.8% during 2013~2016, and declined afterwards to 21.8 in 2019; (2) SSRIs was the second highest prescribed medication with 32.6% utilization rate in 2012, but surpassed benzodiazepine in 2013 (31.2% vs. 26.5%) and became dominate prescribed medication from year 2014–2019 with the utilization rates between 38.4%~41.5%, however slightly declined over the years from 40.3% in 2017 to 38.4% in 2019; (3) Hydroxyzine was the third most used medication for patients with DAD which increased from 11.2% in 2012 to 16.5% in 2019; (4) SNRIs was the fourth most used medication for patients with DAD which increased from 4.1% in 2012 to 12.4% in 2019; (5) Bupropion increased from 5.0% in 2012 to 9.7% in 2019, and TCAs increased from 6.7% in 2012 to 7.8% in 2019; (6) Tetra maintained in 3.02%~3.66% during the year 2012–2019. Overall, the utilization of medication for patients with DAD maintained 61.6%~63.3% during the years 2012–2019 except a drop to 50% in 2013 due to the drop of prescriptions of benzodiazepine (Figure 4).

The utilization of psychotherapy increased from 24.4% in 2012 to 36.5% in 2019, which was mainly contributed from the utilization of individual psychotherapy in Table 3 and Figure 4C2. The utilization of group psychotherapy increased from 2.8% in 2014 to 6.2% in 2019. The utilization of psychotherapy is quite close to the utilization of individual therapy, indicating that almost every patient that received group therapy also participated in individual therapy. The utilization for either medication or psychotherapy maintained in the range of 70.6%~73.4% during 2012–2019 except a dip of 61.8% in 2013 (Figure 4C3). The utilization of medication was slightly decreased, while the utilization of psychotherapy increased over the years from 2012–2019, which caused the increasing utilization of treatment over these years.

### Factors associated with utilization of DAD treatments

We examined the demographic and behavioral factors that could be potentially associated with the utilization of treatment based on the dataset in 2019 using logistic regression models(Table 4 and 5). We found that (1) males had lower utilization of medication (56.8% vs. 63.6%, OR 0.647 and 95%CI (0.636, 0.657)), higher utilization of psychotherapy (39.4% vs. 35.2%, OR 1.114 and 95%CI (1.096, 1.132)), and overall lower utilization of treatment (71.5% vs. 74.2%, OR 0.749 and 95%CI (0.736, 0.763)) than females; (2) the utilization rate of medication increased as age increased from 62.7% at age group 14–24 to the peak utilization rate at 71% at age group 35–54, and dropped to 9.95% for patients of 65 and older; (3) the utilization rate of psychotherapy was 52.4% at age group 14–24, decreased to 13.3% as age increased to 65 and older, which can also be shown from the declined OR as age increased; (4) Non-Hispanic whites patients had the highest utilization of medication (63.5%, followed by 63.3% for non-Hispanic others, 57.3% for Hispanic) and the second highest utilization of any treatment (74.9%, led by non-Hispanic others at 76.8% and followed by Hispanic at 73.9%); (5) patients living in urban areas, comparing with urban area, had higher utilization rate of medication (62.7% vs. 59.9%, OR 1.205 and 95%CI (1.186, 1.225)), lower utilization rate of psychotherapy (35.2% vs. 38.2%, OR 0.965 and 95%CI (0.950, 0.980)), and slightly higher utilization of any treatment (73.9% vs. 72.6%, OR 1.198 and 95%CI (1.177, 1.220)); (6) patients with AUD, comparing without AUD, had higher utilization of medication (70.1% vs. 60.8%, OR 1.438 and 95%CI (1.393, 1.484)), psychotherapy (58.4% vs. 34.9%, OR 2.562 and 95%CI (2.489, 2.637)), and any treatment (83.6% vs. 72.6%, OR 1.893 and 95%CI (1.821, 1.969)); (7) patients with tobacco use, comparing without tobacco use, had higher utilization of medication (64.1% vs. 59.0%, OR 1.079 and 95%CI (1.062, 1.097)), and higher utilization of psychotherapy (37.7% vs. 35.4%, OR 1.129 and 95%CI (1.112, 1.147)).

**Figure 5:**
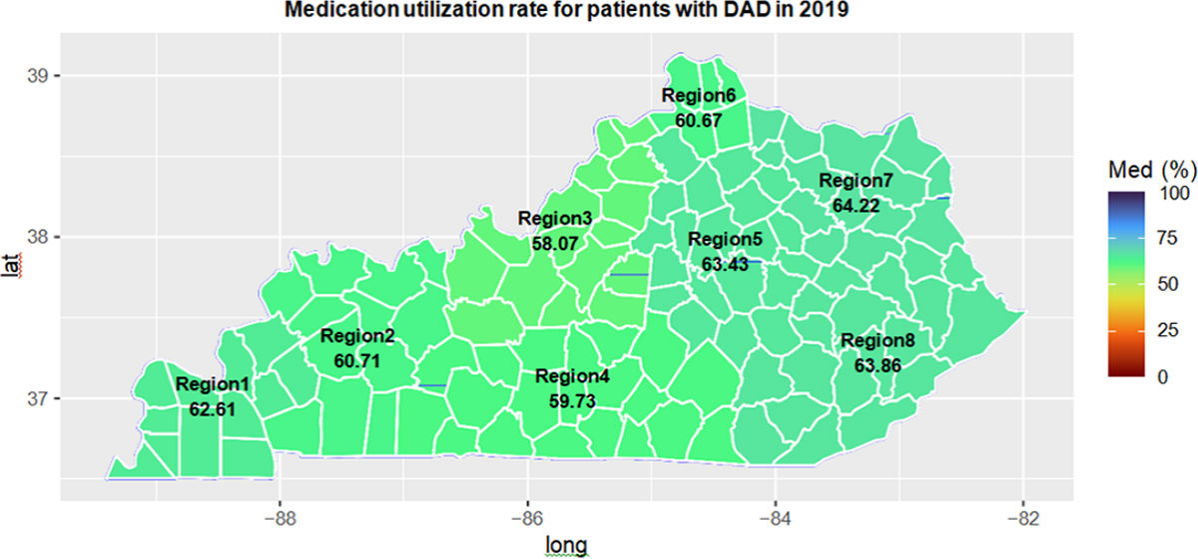
Geographic distribution of treatment utilization for patients with DAD in 2019.

**Figure 6:**
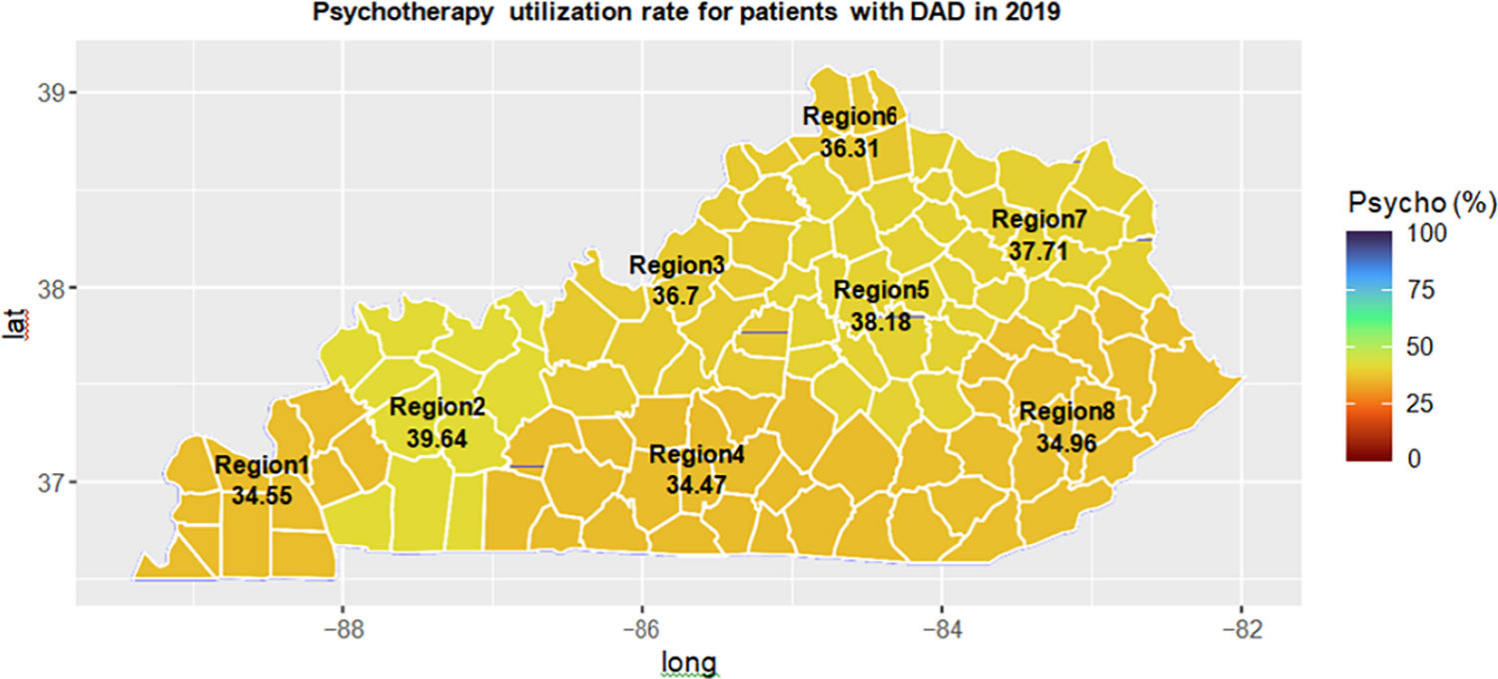
Geographic distribution of treatment utilization for patients with DAD (Panel A2: Psychotherapy utilization rate for patients with DAD in 2019).

We also examined the treatment utilization across the eight different medical regions in Kentucky. Figures 5 and 6 have shown respectively the medication utilization rate and psychotherapy utilization rate over all eight Kentucky medical regions in 2019.

From Figure 5, Region 8 in the eastern Kentucky Appalachian area had the second highest medication utilization rate (63.9%) in 2019, slightly lower than Region 7 (64.2%). However, Region 8 had much higher medication utilization rate (69.6%) in 2012, 7% higher than the second highest one in 2012 (Figure S2). Regional 3 which included Louisville metro had the lowest medication utilization rate in both 2012 and 2019. We also found that the psychotherapy utilization rate has increased over all regions (Figure S2 for 2012 and Figure 6 for 2019). Region 3 (including Louisville metro area) had a higher psychotherapy utilization rate than Region 8 (rural area).

## DISCUSSION

Our study indicated that the prevalence of DAD increased over years, from 30.8% in 2012 to 36.0% in 2019. The prevalence of AUD and tobacco use among patients with DAD was significantly higher than those in the Medicaid population. We found that the prevalence rates of DAD for the northern regions of Kentucky (Region 6) were the highest. Medical region 3 including Louisville metro had the lowest DAD prevalence. The Southeast region of Kentucky (Region 8) had the highest DAD prevalence. Geographical maps for treatment utilization in Figure 5 and Figure S2 and S3 clearly showed the large geographic variation in treatment utilization. Urban areas tended to use less medication but more psychotherapy than rural areas.

Data from this study indicated that patients between 45 and 54 had the highest DAD prevalence across all years 2012–2019, and they were more likely to receive medication than psychotherapy. While the DAD prevalence among patients under 24 was the lowest across all age groups but increased over years, and they had the highest utilization rate of psychotherapy compared with the other age groups. In our study, although patients older than 65 had a higher DAD prevalence rate, they were less likely to receive any treatment for DAD. The group of patients older than 65 were more likely had other comorbid conditions and was treated for these comorbid conditions other than DAD.

More than 60% patients diagnosed with DAD had received pharmacotherapy across eight years from 2012–2019 except in 2013, which dropped significantly due to the reduced utilization of benzodiazepine due to the Kentucky house bill (HB1) on the regulation of pain clinics and prescription drug abuse in Kentucky [13]. Other than 2013, the utilization of pharmacotherapy maintained at about 62%. Utilization of psychotherapy increased over years from 24.4% in 2012 to 36.5% in 2019. Overall, utilization of any treatment slightly increased from 70.9% in 2012 to 73.3% in 2019 except a dip 2013 due to the reduction of benzodiazepine prescription.

Non-Hispanic whites had the highest DAD prevalence than the other race/ethnicity groups. Possible reason for this phenomenon was that both Hispanic and non-Hispanic blacks chose not to visit the mental-health care providers due to the cultural stigma of mental illnesses [14]. However, Hispanic and non-Hispanic blacks had a higher psychotherapy utilization rate than Non-Hispanic whites. This could be attributable to greater concerns among the Hispanic and non-Hispanic blacks patients as regards addiction and ineffectiveness of antidepressants [15].

In contrast to urban areas, rural areas witnessed higher prevalence of DAD (34.2% in urban areas versus 38.3% rural areas) in 2019. Patients with DAD in rural areas tended to seek medication more than psychotherapy, which could reflect the limitation of mental health resources allocated in rural areas [16]. Furthermore, patients with AUD had significantly higher DAD prevalence than patients without AUD (63.3% vs. 38.3%) based on 2019 data, and patients with tobacco use had significantly higher DAD prevalence than patients without tobacco use (50.2% vs. 28.9%). Among patients diagnosed with DAD, patients diagnosed with AUD had higher utilization rates of DAD treatments (70.1% for pharmacotherapy and 58.3% for psychosocial therapy) compared with those without AUD (60.8% for pharmacotherapy and 34.9% for psychosocial therapy); patients with tobacco use had higher utilization rates of DAD treatments (64.1% for pharmacotherapy and 37.7% for psychosocial therapy) compared with those without tobacco use (59.0% for pharmacotherapy and 35.4% for psychosocial therapy). In our previous study, patients with AUD received less than 9% of FDA approved AUD medication [17], however those patients more likely receive DAD related medications. There is a potential for polydrug use for those patients.

There were some limitations to our study. The first was the considerable amount of missing data on race/ethnicity. About 30% of the Medicaid population did not have their information on race/ethnicity available, thus we could not have a more comprehensive assessment on the prevalence of DAD and treatment disparities among different race/ethnicities. Secondly, information on patient-level socioeconomic status was not available. Therefore, we could not assess the impact of socioeconomic status on the prevalence of DAD and treatment utilization. However, we may link the socioeconomic at ZIP code level and county-level from other resources to study the impact of socioeconomic status on the prevalence of DAD and treatment utilization [18,19].

## CONCLUSION

We examined the trends of DAD as well as that of treatment utilization among the Kentucky Medicaid population between 2012 and 2019. We found there was a significant drop of treatment utilization rates in 2013 due to the implementation of the KASPER program with a major reduction in the prescription of benzodiazepine, followed by a steady increase thereafter in both psychotherapies and overall treatments.

The prevalence of DAD was significantly higher among patients with the following characteristics: non-Hispanic whites, females, aged 45–54, resided in the non-metro area, diagnosed with AUD, and used tobaccos. On the other hand, the lowest prevalence of DAD was among patients with the following characteristics: Hispanic, males, younger than 24, resided in the urban area, without AUD, and not using tobaccos. Treatment utilization rate was significantly higher among DAD patients who were non-Hispanic other, females, between 25 and 34 years old, living in rural areas, diagnosed with AUD, and using tobaccos. Treatment utilization was the lowest among patients who were Hispanic, male, older than 65 years old, living in urban area, without AUD, and without using tobaccos.

The overall prevalence of DAD is steadily increasing. The overall treatment utilization for DAD did not change significantly over years, although the components of treatment had changed over years. Comorbid conditions were positively associated with mental health resource utilization among Medicaid enrollees. Health disparities in health care utilization between African Americans and Caucasians still exist and need to be further investigated.

## Supplementary Material

Supplementary Materials

## Figures and Tables

**Table 1:** Prevalence of depressive and anxiety disorders (DAD) and its subcategories over time based on Kentucky Medicaid insured patients who had claims between 01/01/2012 and 12/31/2019.

**Year**	**2012**		**2013**		**2014**		**2015**		**2016**		**2017**		**2018**		**2019**	
**# patients**	**85581**		**85581**		**85581**		**85581**		**85581**		**85581**		**85581**		**85581**	
	**N**	**%**	**N**	**%**	**N**	**%**	**N**	**%**	**N**	**%**	**N**	**%**	**N**	**%**	**N**	**%**
DAD	145367	30.8*	142711	30.8	236020	29.8	278015	31.2	293226	32.0	306954	33.4	313611	34.3	325830	36.3
Anxiety disorder	111893	77.0**	110340	77.3	183484	77.7	218327	78.5	229037	78.1	240813	78.5	247756	79.0	258905	80.1
Depressive disorder	93741	64.5**	91568	64.2	153589	65.1	178782	64.3	174610	59.6	184309	60.0	192208	61.3	202645	62.7
Alcohol use disorder	6939	4.8**	6767	4.7	13578	5.8	17535	6.3	17358	5.9	18062	5.8	20440	6.5	23053	7.1
Tobacco use	53923	37.1**	54311	38.1	98509	41.7	122858	44.1	138761	46.8	148693	48.0	151727	47.9	156649	48.1

Note:

* indicates the percentage of patients diagnosed with DAD among the Medicaid population in that year

** indicates the percentage of patients diagnosed with specified condition among patients with DAD.

**Table 2:** Risk factors for depressive and anxiety disorders (DAD) based on Kentucky Medicaid 2019 database using descriptive statistics as well as multiple logistic regressions.

		Descriptive statistics			Results from logistic regression		
		# patients	# Patients with DAD (%)	P-value	OR	95% CI	P-value
Overall		896695	325830(36.3%)				
Sex	(ref: Female)	530982	222573(41.9%)	<0.001			
	Male	365711	103257(28.2%)		0.484	(0.480, 0.489)	<0.001
Age	(ref: <24)	204485	50582(24.7%)	<0.001			
	[25,34]	185418	62972(34%)		1.222	(1.205, 1.24)	<0.001
	[35,44]	160785	62702(39%)		1.472	(1.45, 1.495)	<0.001
	[45,54]	134219	58595(43.7%)		1.781	(1.754, 1.809)	<0.001
	[55,64]	126207	54319(43%)		1.715	(1.688, 1.743)	<0.001
	>65	85581	36660(42.8%)		1.835	(1.803, 1.868)	<0.001
Race/Ethnicity	(Ref: Non-Hispanic white)	635578	250519(39.4%)	<0.001			
	Hispanic	18137	3491(19.2%)		0.482	(0.464, 0.501)	<0.001
	Non-Hispanic black	90808	21479(23.7%)		0.488	(0.48, 0.497)	<0.001
	Non-Hispanic missing	137060	47245(34.5%)		0.845	(0.834, 0.855)	<0.001
	Non-Hispanic others	15112	3096(20.5%)		0.478	(0.459, 0.498)	<0.001
	Rural	474473	181554(38.3%)		1.085	(1.075, 1.096)	<0.001
	Yes	311879	156649(50.2%)		2.188	(2.166, 2.209)	<0.001
	Yes	36401	23053(63.3%)		2.843	(2.778, 2.909)	<0.001

**Table 3:** Treatment utilization for patients diagnosed with depressive and anxiety disorders (DAD) based on Kentucky Medicaid 2012–2019 database.

	**2012**		**2013**		**2014**		**2015**		**2016**		**2017**		**2018**		**2019**	
**Total patients with DAD**	**145367**		**142711**		**236020**		**278626**		**296289**		**310018**		**316594**		**325830**	
**Treatment**	**N**	**%**	**N**	**%**	**N**	**%**	**N**	**%**	**N**	**%**	**N**	**%**	**N**	**%**	**N**	**%**
Medication	90679	62.38	71332	49.98	145377	61.6	176421	63.32	187182	63.18	193748	62.5	196946	62.21	200265	61.46
SSRIs	47317	32.55	44473	31.16	95700	40.55	115695	41.52	121512	41.01	124822	40.26	125068	39.5	125257	38.44
SNRIs	5953	4.1	6300	4.41	19370	8.21	27036	9.7	31692	10.7	35711	11.52	38349	12.11	40391	12.4
TCAs	9737	6.7	9098	6.38	18071	7.66	21623	7.76	23234	7.84	24893	8.03	25065	7.92	25354	7.78
Tetra	4807	3.31	4311	3.02	7628	3.23	9940	3.57	10478	3.54	11081	3.57	11369	3.59	11939	3.66
Benzo	58613	40.32	37751	26.45	65538	27.77	76311	27.39	78033	26.34	75034	24.2	72307	22.84	71133	21.83
Bupropion	7231	4.97	7183	5.03	16226	6.87	22383	8.03	25795	8.71	28502	9.19	30211	9.54	31461	9.66
Hydroxyzine	16327	11.23	16349	11.46	34303	14.53	42558	15.27	46293	15.62	48843	15.75	51213	16.18	53615	16.45
Psychotherapy	35527	24.44	39545	27.71	61822	26.19	78399	28.14	88665	29.93	99388	32.06	110040	34.76	118956	36.51
IT*	34840	23.97	39004	27.33	61163	25.91	77442	27.79	87151	29.41	97879	31.57	108596	34.3	117453	36.05
GT**	6033	4.15	4653	3.26	6763	2.87	9171	3.29	12044	4.06	14964	4.83	17424	5.5	20267	6.22
Treatment	103013	70.86	88149	61.77	166552	70.57	202294	72.6	215873	72.86	226392	73.03	232352	73.39	238949	73.34

Note:

* IT indicates individual psychotherapy

** GT indicates group psychotherapy.

**Table 4:** Factors associated with treatment utilization for patients with DAD based on Kentucky Medicaid 2019 dataset.

		# Patients with DAD	Any medication		Any psychotherapy		Any treatment	
		N	N	%	N	%	N	%
Overall		325830	200265	61.5	118956	36.5	238949	73.3
Sex	Female	222573	141575	63.6	78276	35.2	165085	74.2
	Male	103257	58689	56.8	40680	39.4	73863	71.5
Age	<24	50582	31697	62.7	26510	52.4	41764	82.6
	[25,34]	62972	43972	69.8	28237	44.8	52350	83.1
	[35,44]	62702	44709	71.3	26394	42.1	51748	82.5
	[45,54]	58595	41678	71.1	19838	33.9	46553	79.5
	[55,64]	54319	34563	63.6	13109	24.1	38671	71.2
	>65	36660	3646	9.95	4868	13.3	7863	21.5
Race/Ethnicity	Non-Hispanic white	250519	158999	63.5	92822	37.1	187716	74.9
	Hispanic	3491	2001	57.3	1457	41.7	2581	73.9
	Non-Hispanic black	21479	11541	53.7	8444	39.3	14986	69.8
	Non-Hispanic missing	47245	25765	54.5	14999	31.8	31288	66.2
	Non-Hispanic others	3096	1959	63.3	1234	39.9	2378	76.8
Geographic region	Urban	144276	86393	59.9	55112	38.2	104763	72.6
	Rural	181554	113872	62.7	63844	35.2	134186	73.9
Tobacco use	No	169181	99875	59	59854	35.4	122452	72.4
	Yes	156649	100390	64.1	59102	37.7	116497	74.4
Alcohol use Disorder	No	302777	184110	60.8	105507	34.9	219674	72.6
	Yes	23053	16155	70.1	13449	58.4	19275	83.6

**Table 5:** Factors associated with treatment utilization based on 2019 Kentucky Medicaid database using multiple logistic regressions.

		Any medication	Any psychotherapy	Any treatment	58.4	58.4	58.4	58.4	58.4	58.4
	
		OR	95% CI	P-Value	OR	95% CI	P-Value	OR	95% CI	P-Value
	
	**Male**	**0.647**	**(0.636, 0.657)**	**<0.001**	**1.114**	**(1.096, 1.132)**	**<0.001**	**0.749**	**(0.736, 0.763)**	**<0.001**

Age (Ref: <24)	[25,34]	1.326	(1.293, 1.36)	<0.001	0.684	(0.668, 0.701)	<0.001	1.012	(0.981, 1.045)	0.442

	[35,44]	1.417	(1.381, 1.454)	<0.001	0.597	(0.583, 0.612)	<0.001	0.963	(0.933, 0.994)	0.02

	[45,54]	1.402	(1.366, 1.439)	<0.001	0.419	(0.409, 0.43)	<0.001	0.786	(0.762, 0.811)	<0.001

	[55,64]	0.996	(0.97, 1.022)	0.738	0.26	(0.253, 0.268)	<0.001	0.505	(0.49, 0.52)	<0.001

	>65	0.061	(0.058, 0.063)	<0.001	0.137	(0.133, 0.142)	<0.001	0.055	(0.053, 0.057)	<0.001

Race/Ethnicity (Ref: Non-Hispanic White)	Hispanic	0.773	(0.719, 0.83)	<0.001	1.032	(0.962, 1.108)	0.38	0.837	(0.771, 0.909)	<0.001

	Non-Hispanic black	0.609	(0.591, 0.628)	<0.001	0.992	(0.962, 1.022)	0.59	0.671	(0.649, 0.694)	<0.001

	Non-Hispanic missing	0.867	(0.848, 0.887)	<0.001	0.893	(0.873, 0.913)	<0.001	0.843	(0.823, 0.864)	<0.001

	Non-Hispanic other	0.945	(0.875, 1.022)	0.153	0.921	(0.854, 0.992)	0.03	0.935	(0.854, 1.024)	0.142

Geographic Region	Rural	1.205	(1.186, 1.225)	<0.001	0.965	(0.95, 0.98)	<0.001	1.198	(1.177, 1.22)	<0.001

Tobacco Use	Yes	1.079	(1.062, 1.097)	<0.001	1.129	(1.112, 1.147)	<0.001	0.985	(0.967, 1.002)	0.089

Alcohol Use Disorder	Yes	1.438	(1.393, 1.484)	<0.001	2.562	(2.489, 2.637)	<0.001	1.893	(1.821, 1.969)	<0.001
