# Supplementary material for: Prevalence and Treatment Utilization of Patients Diagnosed with Depression and Anxiety Disorders Based on Kentucky Medicaid 2012–2019 Datasets
Source: J Depress Anxiety. Author manuscript; Available in PMC 2023 Aug 15. (PMC10427139)

Supplementary Materials for the manuscript “Prevalence and treatment patterns for depressive and anxiety disorders (DAD) based on Kentucky Medicaid 2012-2019 datasets”.

**Table S1:** ICD-9 and ICD-10 diagnosis codes for depressive and anxiety disorders (DAD) and other Comorbidities.

| Condition | ICD-9 Codes | ICD-10 Codes |
| --- | --- | --- |
| Major depressive disorders | 296.2x, 296.3x,  300.4, 311 | F32.x, F33.x, F34.1 |
| Anxiety disorders | 293.84, 300.x,  309.81 | F06.4, F40.x, F41.x,  F42.x, F44.9, F45.5,  F45.6, F45.7, F45.8,  F48.8, F48.9, F99, R45.2 |
| **Alcohol use disorder** | | |
| Nondependent Abuse | 305.x | F101.x |
| Alcohol dependence | 303.x | F102.x |
|  | | |
| **Tobacco use** | 305.1, V15.82 | F17.x, Z71.6, Z72.0 |

\

**Table S2:** Classes of medications for treating patients with depression and anxiety disorders (DAD)

| Drug Class | Prescription | Notes |
| --- | --- | --- |
| (1) Selective Serotonin Reuptake Inhibitors  (SSRIs) | Fluoxetine, Citalopram, Sertraline,  Paroxetine, Escitalopram | For both depressive and anxiety disorders |
| (2) Serotonin-Norepinephrine Reuptake Inhibitors  (SNRIs) | Venlafaxine, Duloxetine, Desvenlafaxine |  |
| (3) Tricyclic Antidepressants  (TCAs) | Amitriptyline, Amoxapine, Clomipramine,  Desipramine, Doxepin, Imipramine,  Nortriptyline, Protriptyline, Trimipramine |  |
| (4) Tetracyclic Antidepressants | Mirtazapine | Mainly for Depressive disorder |
| (5) Benzodiazepines | Diazepam, Clonazepam, Lorazepam,  Alprazolam | Mainly for anxiety disorders |
| (6) Bupropion | Bupropion | For both depressive and anxiety disorders |
| (7) Hydroxyzine | Hydroxyzine |  |

**Table S3:** Current Procedural Terminology (CPT) codes for psychotherapy treatments

|  | CPT code |
| --- | --- |
| Individual Psychotherapy (IT) | 90785,90791,90792,  90804,90806,90808,90810,90811,90812,90814, 90832,90833,90834,90836,90837,90838,90839,90840, 90845,  90875,90876,  96152,96154,96155 |
| Group Psychotherapy (GT) | 90846,90847,90849,  90853,90857,96153 |

**Table S4:** Risk factors for depressive and anxiety disorders (DAD) based on Kentucky Medicaid 2012 database

|  |  | Descriptive statistics | | | Results from logistic regression | | |
| --- | --- | --- | --- | --- | --- | --- | --- |
|  |  | N | n(%) | P-value | OR | 95% CI | P-Value |
| Overall |  | 471415 | 145367(30.8%) |  |  |  |  |
| Sex | (ref: Female) | 308909 | 103860(33.6%) | <0.001 |  |  |  |
|  | Male | 162506 | 41507(25.5%) |  | 0.59 | (0.581, 0.599) | <0.001 |
| Age | (ref: <24) | 134512 | 19904(14.8%) | <0.001 |  |  |  |
|  | [25,34] | 80163 | 23214(29%) |  | 1.884 | (1.843, 1.927) | <0.001 |
|  | [35,44] | 60920 | 25254(41.5%) |  | 3.258 | (3.184, 3.333) | <0.001 |
|  | [45,54] | 62266 | 28822(46.3%) |  | 3.893 | (3.805, 3.982) | <0.001 |
|  | [55,64] | 56120 | 23715(42.3%) |  | 3.377 | (3.298, 3.457) | <0.001 |
|  | >65 | 77434 | 24458(31.6%) |  | 2.33 | (2.279, 2.381) | <0.001 |
| Race  /Ethnicity | (ref: Non-Hispanic White) | 327380 | 107691(32.9%) | <0.001 |  |  |  |
|  | Hispanic | 5590 | 694(12.4%) |  | 0.408 | (0.376, 0.443) | <0.001 |
|  | Non-Hispanic Black | 43072 | 7860(18.2%) |  | 0.492 | (0.479, 0.506) | <0.001 |
|  | Non-Hispanic  Missing | 84233 | 27465(32.6%) |  | 0.907 | (0.891, 0.923) | <0.001 |
|  | Non-Hispanic Other | 11140 | 1657(14.9%) |  | 0.477 | (0.452, 0.504) | <0.001 |
| Geographic region | (ref: Metro) | 197180 | 55137(28.0%) | <0.001 |  |  |  |
|  | Non-Metro | 274235 | 90230(32.9%) |  | 1.126 | (1.11, 1.142) | <0.001 |
| Tobacco use | (ref: No) | 365699 | 91444(25.0%) | <0.001 |  |  |  |
|  | Yes | 105716 | 53923(51%) |  | 2.451 | (2.414, 2.488) | <0.001 |
| Alcohol use disorder | (ref: No) | 459832 | 138428(30.1%) | <0.001 |  |  |  |
|  | Yes | 11583 | 6939(59.9%) |  | 2.315 | (2.223, 2.411) | <0.001 |

Table S5: Descriptive characteristics of Mental Disorder cohorts based on Kentucky Medicaid Data from 01/01/2012 to 12/31/2018.

|  |  | 2012 | | 2013 | | 2014 | | 2015 | | 2016 | | 2017 | | 2018 | |
| --- | --- | --- | --- | --- | --- | --- | --- | --- | --- | --- | --- | --- | --- | --- | --- |
| Total |  | 471415 | 145367(30.8%) | 463989 | 142711(30.8%) | 791575 | 236020(29.8%) | 891448 | 278015(31.2%) | 916002 | 293226(32%) | 919921 | 306954(33.4%) | 915672 | 313611(34.2%) |
| Sex | F | 308909 | 103860(33.6%) | 303105 | 101903(33.6%) | 476051 | 161774(34%) | 527051 | 189164(35.9%) | 540563 | 199797(37%) | 542919 | 209793(38.6%) | 540711 | 214141(39.6%) |
|  | M | 162506 | 41507(25.5%) | 160884 | 40808(25.4%) | 315523 | 74246(23.5%) | 364396 | 88851(24.4%) | 375438 | 93429(24.9%) | 377001 | 97161(25.8%) | 374960 | 99469(26.5%) |
| Age | <24 | 134512 | 19904(14.8%) | 131456 | 18866(14.4%) | 180203 | 28673(15.9%) | 199776 | 36758(18.4%) | 203880 | 40190(19.7%) | 205925 | 43553(21.1%) | 208351 | 47258(22.7%) |
|  | [25,34] | 80163 | 23214(29%) | 77645 | 21363(27.5%) | 164529 | 43039(26.2%) | 191047 | 52613(27.5%) | 195692 | 56394(28.8%) | 195079 | 59426(30.5%) | 192417 | 60514(31.4%) |
|  | [35,44] | 60920 | 25254(41.5%) | 59659 | 24324(40.8%) | 134919 | 46782(34.7%) | 156877 | 55657(35.5%) | 162300 | 58397(36%) | 163873 | 60884(37.2%) | 164123 | 61442(37.4%) |
|  | [45,54] | 62266 | 28822(46.3%) | 60299 | 27861(46.2%) | 128224 | 50911(39.7%) | 144495 | 58294(40.3%) | 146431 | 60325(41.2%) | 144103 | 60624(42.1%) | 139764 | 59537(42.6%) |
|  | [55,64] | 56120 | 23715(42.3%) | 57667 | 24694(42.8%) | 103280 | 39214(38%) | 117671 | 45427(38.6%) | 123694 | 48999(39.6%) | 126587 | 51707(40.8%) | 126385 | 52919(41.9%) |
|  | >65 | 77434 | 24458(31.6%) | 77263 | 25603(33.1%) | 80420 | 27401(34.1%) | 81582 | 29877(36.6%) | 84005 | 31984(38.1%) | 84354 | 33824(40.1%) | 84632 | 34924(41.3%) |
| Race  /Ethnicity | Hispanic | 5590 | 694(12.4%) | 5848 | 691(11.8%) | 9404 | 1432(15.2%) | 12092 | 1967(16.3%) | 15143 | 2309(15.2%) | 16719 | 2789(16.7%) | 17485 | 3119(17.8%) |
|  | Non.Hispanic.Black | 43072 | 7860(18.2%) | 42143 | 7522(17.8%) | 71225 | 12579(17.7%) | 82309 | 15555(18.9%) | 87911 | 16970(19.3%) | 90572 | 18833(20.8%) | 92191 | 20174(21.9%) |
|  | Non.Hispanic.Missing | 84233 | 27465(32.6%) | 85816 | 28128(32.8%) | 128569 | 38913(30.3%) | 154500 | 47462(30.7%) | 154454 | 47848(31%) | 142609 | 45102(31.6%) | 138740 | 45010(32.4%) |
|  | Non.Hispanic.Other | 11140 | 1657(14.9%) | 11628 | 1801(15.5%) | 46473 | 8918(19.2%) | 31299 | 4693(15%) | 13862 | 2154(15.5%) | 14205 | 2453(17.3%) | 14807 | 2739(18.5%) |
|  | Non.Hispanic.White | 327380 | 107691(32.9%) | 318554 | 104569(32.8%) | 535904 | 174178(32.5%) | 611248 | 208338(34.1%) | 644632 | 223945(34.7%) | 655816 | 237777(36.3%) | 652449 | 242569(37.2%) |
| Geographic region | metro | 197180 | 55137(28%) | 194451 | 53982(27.8%) | 354731 | 98060(27.6%) | 411567 | 119109(28.9%) | 425417 | 126615(29.8%) | 428700 | 133374(31.1%) | 429110 | 138220(32.2%) |
|  | nonmetro | 274235 | 90230(32.9%) | 269538 | 88729(32.9%) | 436844 | 137960(31.6%) | 479881 | 158906(33.1%) | 490585 | 166611(34%) | 491221 | 173580(35.3%) | 486562 | 175391(36%) |
| Tobacco.use | NO | 365699 | 91444(25%) | 356949 | 88400(24.8%) | 573425 | 137511(24%) | 631035 | 158577(25.1%) | 651582 | 172333(26.4%) | 646906 | 179148(27.7%) | 644361 | 183980(28.6%) |
|  | YES | 105716 | 53923(51%) | 107040 | 54311(50.7%) | 218150 | 98509(45.2%) | 260413 | 119438(45.9%) | 264420 | 120893(45.7%) | 273015 | 127806(46.8%) | 271311 | 129631(47.8%) |
| AUD | NO | 459832 | 138428(30.1%) | 452789 | 135944(30%) | 767907 | 222442(29%) | 861852 | 260499(30.2%) | 886328 | 275957(31.1%) | 889864 | 289023(32.5%) | 882376 | 293297(33.2%) |
|  | YES | 11583 | 6939(59.9%) | 11200 | 6767(60.4%) | 23668 | 13578(57.4%) | 29596 | 17516(59.2%) | 29674 | 17269(58.2%) | 30057 | 17931(59.7%) | 33296 | 20314(61%) |
| Med region | MED_Region1 | 23060 | 6968(30.2%) | 22533 | 6730(29.9%) | 38737 | 11682(30.2%) | 43919 | 13653(31.1%) | 45395 | 14485(31.9%) | 46307 | 15500(33.5%) | 46545 | 16605(35.7%) |
|  | MED_Region2 | 40373 | 11955(29.6%) | 39505 | 12281(31.1%) | 64822 | 19351(29.9%) | 74802 | 22960(30.7%) | 77563 | 24561(31.7%) | 78212 | 26130(33.4%) | 78117 | 26634(34.1%) |
|  | MED_Region3 | 100666 | 25132(25%) | 99052 | 23987(24.2%) | 182425 | 47625(26.1%) | 211237 | 58409(27.7%) | 217518 | 61354(28.2%) | 218969 | 65486(29.9%) | 219140 | 67627(30.9%) |
|  | MED_Region4 | 65923 | 21028(31.9%) | 64652 | 20809(32.2%) | 107467 | 32956(30.7%) | 119453 | 37976(31.8%) | 121892 | 38945(32%) | 122556 | 39944(32.6%) | 122312 | 40850(33.4%) |
|  | MED_Region5 | 76143 | 23126(30.4%) | 75546 | 22328(29.6%) | 136635 | 37425(27.4%) | 155579 | 45875(29.5%) | 160489 | 50722(31.6%) | 161310 | 53159(33%) | 161296 | 54709(33.9%) |
|  | MED_Region6 | 27424 | 10037(36.6%) | 26977 | 9924(36.8%) | 50013 | 17236(34.5%) | 57728 | 19903(34.5%) | 59470 | 20568(34.6%) | 59775 | 20937(35%) | 59287 | 21607(36.4%) |
|  | MED_Region7 | 35543 | 11152(31.4%) | 34796 | 11017(31.7%) | 57205 | 17517(30.6%) | 62885 | 20282(32.3%) | 64462 | 21211(32.9%) | 64981 | 22439(34.5%) | 64395 | 23141(35.9%) |
|  | MED_Region8 | 102269 | 35964(35.2%) | 100923 | 35633(35.3%) | 154249 | 52223(33.9%) | 165839 | 58957(35.6%) | 169209 | 61378(36.3%) | 167807 | 63359(37.8%) | 164578 | 62438(37.9%) |

**Figure S1:** Geographic distribution of prevalence of DAD in 2012


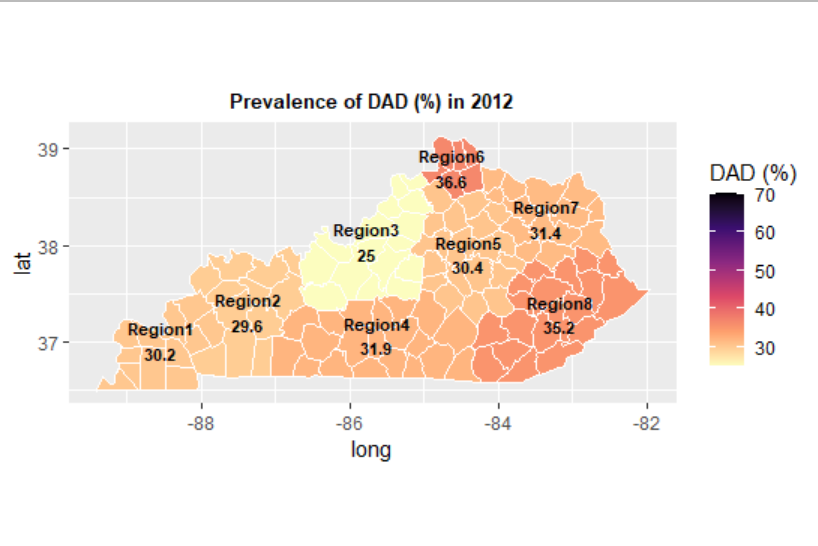


**Figure S2**: Geographic distribution of treatment utilization for patients with DAD based on Kentucky Medicaid data 2012 (Panel A1: Medication utilization rate for patients with DAD in 2012; Panel A2: Psychotherapy utilization rate for patients with DAD in 2012)


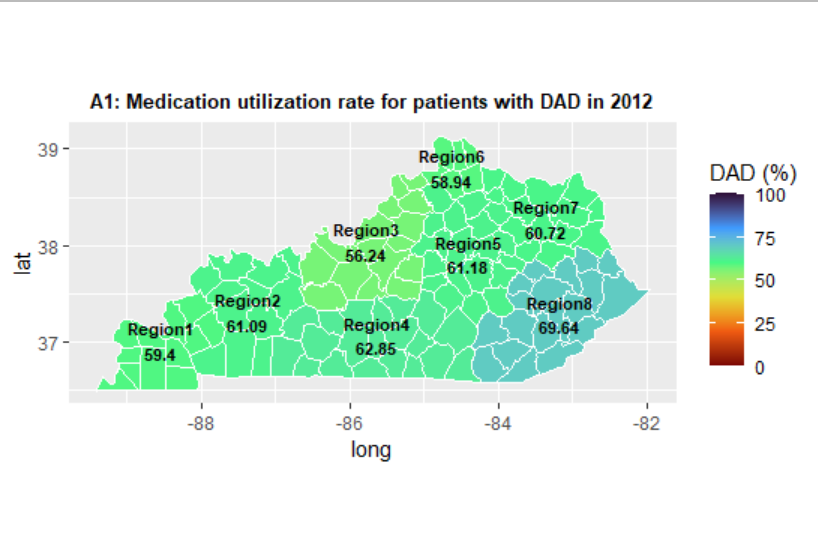


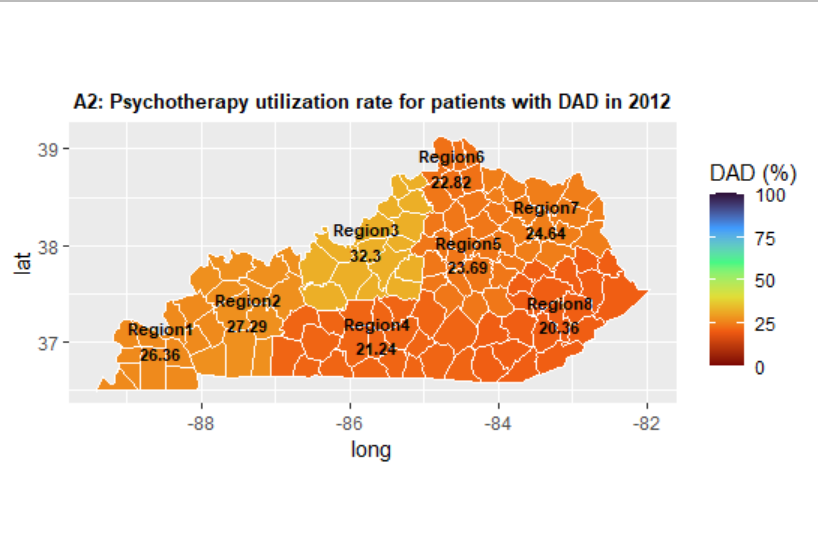

Supplement: Supplementary Materials [file NIHMS1858220-supplement-Supplementary_Materials.docx]
